# Early Postoperative Evaluation of Arthrogenic Muscle Inhibition, Anterior Knee Laxity, and Kinesiophobia After ACL Reconstruction: A Cross-Sectional Observational Study

**DOI:** 10.3390/healthcare13131481

**Published:** 2025-06-20

**Authors:** Florian Forelli, Yoann Demangeot, Agathe Dourver, Adrien Cerrito

**Affiliations:** 1 Haute-Ecole Arc Santé, HES-SO University of Applied Sciences and Arts Western Switzerland, 2800 Delémont, Switzerland; adrien.cerrito@he-arc.ch; 2 Orthopaedic Surgery Department, Clinic of Domont, Ramsay Healthcare, @OrthoLab, 95330 Domont, France; 3 Société Française des Masseurs—Kinésithérapeutes du Sport Lab, 93380 Pierrefite sur Seine, France; 4 MATIM, University of Reims Champagne-Ardenne, 51100 Reims, France; ydemangeot@chu-reims.fr; 5 Therapeutic and Performance Sports Institute, MotionLab, 1052 Le Mont-sur-Lausanne, Switzerland; 6 Faculty of Biology and Medicine, University of Lausanne, 1005 Lausanne, Switzerland; 7 Santy Paramedical Centre, 4 rue Jean Sarrazin, 69008 Lyon, France; agathe.dourver@hotmail.fr

**Keywords:** arthrogenic muscle inhibition, anterior cruciate ligament reconstruction, kinesiophobia, knee laxity, early rehabilitation

## Abstract

Background: Arthrogenic muscle inhibition (AMI), anterior knee laxity, and kinesiophobia are key barriers to recovery after anterior cruciate ligament reconstruction (ACLR). While each has been independently studied, their interrelationships during the early postoperative phase remain unclear. Methods: This cross-sectional study included 56 patients (mean age: 26.5 ± 5.7 years) who underwent ACLR using hamstring autografts. Clinical AMI grading, GNRB^®^ arthrometer measurements of anterior tibial translation, and the Tampa Scale for Kinesiophobia-11 (TSK-11) were used to assess neuromuscular inhibition, mechanical laxity, and psychological fear, respectively. All evaluations were performed at 34.9 ± 4.2 postoperative days. Statistical analyses included one-way ANOVA, Kruskal–Wallis, and Spearman correlation. Results: No significant differences in TSK-11 scores were observed across AMI grades (*p* = 0.327). Similarly, anterior laxity did not differ significantly between AMI groups (*p* = 0.182). Correlation between GNRB measurements and TSK-11 scores was non-significant (rho = −0.220, *p* = 0.103). Conclusions: In the early phase following ACLR, AMI, anterior laxity, and kinesiophobia appear to be independent domains. These findings suggest that early postoperative rehabilitation should address each dimension individually. Further longitudinal studies are needed to explore their potential interactions over time.

## 1. Introduction

Anterior cruciate ligament (ACL) injuries are among the most common and debilitating musculoskeletal conditions in athletic populations [1,2]. Incidence rates exceed 100 per 100,000 people-years in young athletes, particularly those involved in pivoting sports [3]. Over 70% of ACL tears result from non-contact mechanisms, such as cutting or deceleration maneuvers, and frequently require surgical reconstruction to restore stability and functional performance [4,5,6]. ACL reconstruction (ACLR) using hamstring tendon autografts is considered a safe and reliable procedure, with high rates of mechanical graft stability and good short- to mid-term outcomes [7]. However, up to 35–45% of patients fail to return to their pre-injury level of sport at one year postoperatively, despite technically successful surgery [8,9,10].

Physiologically, ACL ruptures most commonly result from non-contact actions combining excessive anterior tibial translation, dynamic knee valgus, and internal rotation—especially during abrupt deceleration, pivoting, or landing maneuvers [11]. These biomechanical forces exceed the ligament’s tensile limits, causing structural failure.

Neurophysiologically, ACL injury disrupts proprioceptive afferents from mechanoreceptors within the ligament, leading to altered sensory feedback, impaired motor planning, and neuroplastic changes [12,13]. Studies report deficits in reaction speed, processing time, proprioception, and neuromuscular control—which often persist long after reconstruction [14,15]. These neurocognitive and neurophysiological alterations may predispose to inefficient movement patterns and increase the risk of re-injury, highlighting the importance of integrating early rehabilitation strategies that target both physical and cognitive control systems [16].

This gap between structural repair and functional recovery underscores the multifactorial nature of ACLR rehabilitation. One of the primary neuromuscular barriers is arthrogenic muscle inhibition (AMI), a reflex-mediated inhibition of quadriceps motor unit recruitment triggered by joint effusion, inflammation, and altered sensory feedback [17,18,19]. AMI compromises voluntary muscle activation and strength development, prolonging recovery and increasing the risk of secondary injury [20]. It can persist for several months postoperatively and has been strongly associated with lower quadriceps torque and reduced performance on return-to-sport tests [19,21]. Although the early inflammatory phase may amplify AMI to joint effusion and pain, assessing patients during this window is critical to identify and address early neuromuscular deficits that can hinder rehabilitation.

Alongside neuromuscular inhibition, mechanical parameters such as anterior knee laxity continue to be key metrics for evaluating ACLR success. Arthrometric devices like the KT-1000 and GNRB quantify tibial translation under controlled loads and provide objective data on graft integrity [22,23,24,25,26]. While many patients show satisfactory anterior stability after ACLR, even mild residual laxity has been linked to altered proprioception and potentially to impaired motor coordination [27]. However, other studies report no significant differences in quadriceps strength or functional outcomes across varying degrees of residual laxity, suggesting a complex and potentially indirect relationship [28].

More recently, the role of psychological readiness—especially kinesiophobia—has received growing attention in ACLR literature [10,29,30]. Defined as the irrational and excessive fear of movement due to anticipated pain or reinjury [20], kinesiophobia can severely limit patient engagement in rehabilitation and delay return to sport [10,31,32]. High Tampa Scale for Kinesiophobia (TSK) scores have been associated with reduced strength, lower hop test scores, and lower functional knee scores [33]. Despite its importance, psychological factors are rarely evaluated systematically in the early phases of rehabilitation, representing a significant gap in current clinical practice.

This early postoperative phase—between the 4th and 6th week—is particularly critical, as it represents a window of heightened neuroplasticity and behavioral adaptability [26,27]. During this period, neuromuscular deficits such as AMI are predominantly influenced by intra-articular inflammation and altered sensory feedback [8,10], while psychological responses like kinesiophobia may begin to emerge as patients transition from passive healing to more active rehabilitation [20,22]. Early evaluation during this window may thus allow clinicians to identify recovery barriers at a stage when they are most modifiable.

Although AMI, anterior laxity, and kinesiophobia have each been individually studied, the interactions among them remain poorly understood, particularly during the early postoperative period. From a theoretical standpoint, increased anterior laxity could exacerbate AMI through disruption of proprioceptive input. In turn, persistent AMI may limit motor performance and reinforce psychological fear of movement, thereby increasing kinesiophobia [14,19,21]. This feedback loop may hinder progression through key stages of rehabilitation, particularly during the critical 4–6 week window when neuromuscular plasticity and behavioral adaptation are most responsive to intervention [34,35].

Despite these conceptual links, few studies have concurrently examined mechanical, neuromuscular, and psychological domains in the same early postoperative cohort [14,36,37]. Understanding how these variables interact may reveal early markers of delayed recovery and identify patients at risk for persistent functional deficits. More importantly, it may support the design of integrated rehabilitation strategies that combine biomechanical reconditioning with neuromuscular facilitation and psychological desensitization.

The present study was conducted to explore the relationships between AMI (as assessed clinically), anterior tibial translation (as measured by the GNRB arthrometer), and kinesiophobia (as measured by the TSK-11) in patients assessed between 4 and 6 weeks following ACLR using hamstring autografts. By investigating these parameters together, this study seeks to offer a more holistic understanding of the barriers to early functional recovery.

## 2. Materials and Methods

### 2.1. Study Design

This cross-sectional observational study was designed as a retrospective observational analysis with the aim of investigating the relationships between AMI, anterior knee laxity, and kinesiophobia in the early postoperative period after ACLR.

The study was approved by the Ramsay Healthcare ethics committee and conducted in accordance with the Declaration of Helsinki. All participants provided written informed consent before inclusion.

### 2.2. Participants

Patients were recruited based on strict eligibility criteria. To be included, patients had to be aged between 18 and 35 years, have a body mass index below 30 kg/m^2^, and present a non-contact ACL injury that occurred during sports activity. All patients underwent primary ACLR using a standardized arthroscopic technique with hamstring autografts, without any extra-articular augmentation, performed by one of three experienced knee surgeons (10 years). All participants were physically active individuals engaged in regular recreational or competitive pivoting sports with a high pre-injury activity level—defined by a Tegner Activity Scale score of at least 6 [38] and a Marx Activity Score of at least 6 [39]—were considered eligible. In addition, all participants were evaluated between the 4th and 6th postoperative weeks. All participants followed a standardized rehabilitation protocol post-ACLR, which included progressive phases of range of motion, strengthening, and functional exercises supervised by experienced physical therapists. While adherence was encouraged and monitored, variability in individual compliance may represent a potential source of bias.

Patients were excluded if they had any previous or concurrent injury or surgery to the ipsilateral knee, underwent ACL revision surgery, or presented with associated osteochondral lesions or multiligament injuries. However, patients with meniscal lesions treated by repair or meniscectomy were not excluded.

### 2.3. Sample Size Calculations

A priori sample size estimation was performed using G*Power software (version 3.1.9.7, Universität Düsseldorf, Düsseldorf, Germany) to determine the number of participants required to detect a statistically significant correlation between the primary variables: AMI, anterior tibial laxity, and kinesiophobia scores. Based on an expected moderate correlation coefficient (r = 0.40), an alpha level of 0.05, and a desired power of 0.80, the minimum sample size required was calculated to be 47 participants. This estimation was derived using a two-tailed test for bivariate correlation. Assuming 10% missing data, a total of 53 participants were required for this study.

### 2.4. Outcome Measures

All assessments were conducted by a licensed physical therapist with over 5 years of experience in sports rehabilitation. Evaluations were performed in a controlled clinical setting, following a standardized sequence for all participants, at approximately 4–6 weeks postoperatively (34.9 days ± 4.2). All clinical and functional assessments were conducted during a single session.

AMI was assessed using a standardized clinical grading scale based on active quadriceps recruitment and the presence or absence of full knee extension, as previously described [8,9,10,33]. The clinical grading of AMI was performed by a single licensed physical therapist with more than 5 years of experience in musculoskeletal evaluation and ACL rehabilitation. While this scale has been used in prior studies, it lacks formal validation or inter-rater reliability testing. As such, it represents a semi-objective measure, which may introduce variability in the assessment of AMI severity. The examination was conducted with the patient in a supine position and included visual inspection, palpation, and manual resistance testing. This clinical scale provides a practical method for identifying neuromuscular inhibition, as previously described in the literature [17,18,19,40].

Anterior knee laxity was measured using the GNRB^®^ arthrometer (Genourob, France), a validated and reproducible instrument for quantifying anterior tibial translation [23]. The test was performed with the patient in a supine position and the knee flexed to 20°. A standardized anterior tibial load of 134 N was applied, and the displacement (in millimeters) was recorded [25,26,41,42,43]. The difference in millimeters between the operated and the contralateral knee was used as the main variable of interest for mechanical laxity.

Kinesiophobia was evaluated using the TSK in its 11-item version (TSK-11). This self-administered questionnaire assesses fear of movement and reinjury, with each item rated on a 4-point Likert scale. The total score ranges from 11 to 44, with higher scores indicating greater kinesiophobia [44]. Patients completed the questionnaire independently during the same clinical session.

**Figure 1:**
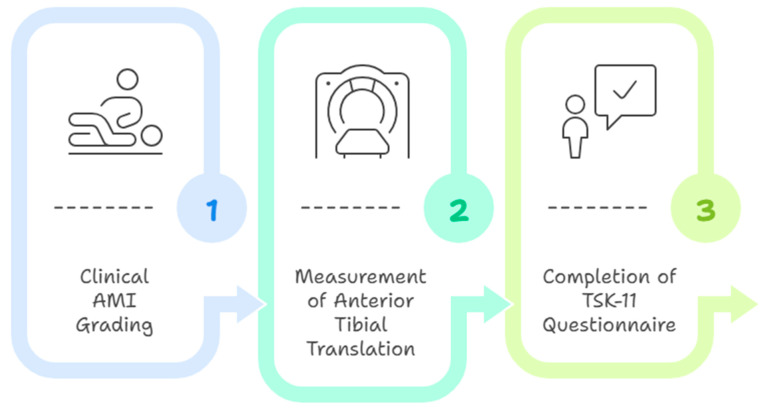
Evaluation Procedure.

The evaluation was conducted in three successive stages: (1) clinical AMI grading through observation of quadriceps contraction and knee extension capacity in a supine position; (2) measurement of anterior tibial translation using the GNRB arthrometer (intra- and inter-rater reliability coefficients ranging from 0.72 to 0.83 depending on load level; standard error of measurement between 3.47 and 3.76 mm per newton; minimal detectable change, approximately 9.6 mm per newton; test–retest and inter-operator reliability coefficients up to 0.99; and agreement limits within ±7.6 percent in subjects with anterior cruciate ligament injury [23]), with participants in a relaxed supine position and the knee at 20° flexion; (3) completion of the TSK-11 questionnaire (internal consistency: Cronbach’s α ≈ 0.79; test–retest reliability: Intraclass Correlation Coefficient ≈ 0.81; standard error of measurement: ≈2.5 points; two-factor structure: activity avoidance and somatic focus [45]) by the participant in a seated position [29,33], supervised by the evaluator to clarify any comprehension issues (Figure 1).

### 2.5. Statistical Analysis

Statistical analyses were performed using JASP software (version 0.19.3, Amsterdam, The Netherlands), with an alpha set to 0.05. Patient characteristics were analyzed with descriptive statistics (age, height, weight, and body mass index). Normality of the data obtained for TSK and GNRB was assessed using the Shapiro–Wilk test. Then the association between TSK and GNRB was assessed using Pearson’s correlation coefficient or Spearman’s rank correlation for non-parametric data. Correlations were considered as negligible (0.0–0.3), low (0.3–0.5), moderate (0.5–0.7) high (0.7–0.9), and very high (0.9–1.0) [46].

To assess the relationship between AMI grades and both TSK and GNRB results, a one-way ANOVA (or a Kruskal–Wallis H test if the one-way ANOVA assumptions were not met) was performed to test for differences in TSK scores and GNRB values as a function of the grade of AMI. Homogeneity of variances was assessed using Levene’s test for equality of variances.

## 3. Results

### 3.1. Participants

The study included 56 participants (41 men and 15 women) with a mean age of 26.48 ± 5.71 years. The mean height was 175.88 ± 9.79 cm, mean weight 81.16 ± 19.66 kg, and mean body mass index 26.08 ± 5.00 kg/m^2^ (Table 1).

### 3.2. Kinesiophobia

**Figure 2:**
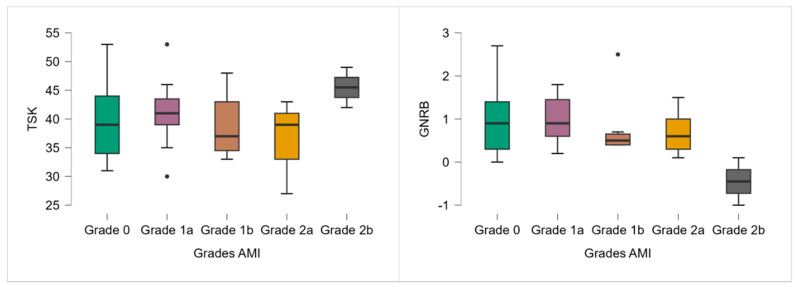
TSK-11 and GNRB results according to the AMI grade. Note: ● *p* < 0.05.

Although no significant differences in TSK-11 scores were observed across AMI grades (*p* = 0.327), a trend toward higher scores was noted in the most severe AMI group (grade 2b), which had a mean TSK-11 of 45.50 (Table 2; Figure 2).

### 3.3. Anterior Knee Laxity

Similarly, although the Kruskal–Wallis test revealed no significant differences in anterior knee laxity between AMI grades (*p* = 0.182), a decreasing trend in GNRB values was observed with increasing AMI severity (Table 2; Figure 2). Grade 2b participants even demonstrated a negative side-to-side difference (−0.45 mm). 

### 3.4. Correlation Between Kinesiophobia and Anterior Knee Laxity

No significant correlation was found between kinesiophobia (TSK-11) and anterior knee laxity (GNRB) at 1 month postoperative (rho = −0.220; *p* = 0.103; Spearman correlation).

## 4. Discussion

The aim of this study was to investigate the early postoperative relationships between AMI, anterior knee laxity (as assessed using the GNRB arthrometer), and kinesiophobia (measured by the TSK-11) in patients 1 month after ACLR with hamstring autografts. Our findings indicate no statistically significant association between these three variables at this early stage of rehabilitation.

In the present study, no significant differences in TSK-11 scores were observed across varying grades of AMI. This result suggests that, during the early postoperative period (1 month after ACLR), clinically assessed AMI does not appear to be associated with increased levels of kinesiophobia. This finding is particularly notable, as it runs counter to the hypothesis that neuromuscular dysfunction—especially quadriceps inhibition—would contribute to psychological barriers such as fear of movement or reinjury. Evaluating patients during the early postoperative inflammatory phase likely increases the detection of AMI due to acute joint swelling, pain, and associated neural reflex inhibition. While this timing may bias the prevalence and severity of AMI observed, it offers valuable insight into the initial neuromuscular barriers that patients face in rehabilitation. This critical window allows clinicians to identify and target impairments that could hinder recovery if left unaddressed.

Theoretically, AMI reduces voluntary quadriceps activation through a reflex inhibition mechanism triggered by joint effusion, nociception, and altered proprioception [17,47]. This diminished motor control could be expected to negatively impact perceived function and, by extension, increase fear-avoidant behaviors [48,49]. However, our findings did not support this relationship in the early postoperative phase. Several explanations may account for this discrepancy. First, previous studies that identified associations between neuromuscular deficits and psychological outcomes often examined patients at later stages of rehabilitation [50,51]. For instance, Isaji et al. found that higher TSK-11 scores at six months after ACLR correlated with poorer knee function and strength measures [33]. Similarly, Burland et al. reported that fear of reinjury and kinesiophobia were significant barriers to return to sport in the months following surgery, often persisting well beyond structural healing [31]. In contrast, the current study’s 4–6-week time window may be too early for the psychological consequences of AMI to fully manifest, particularly given that patients are still undergoing supervised rehabilitation and are generally not yet facing high-stakes functional challenges or return-to-sport decisions.

Second, the measurement modalities may influence the observed relationship. The clinical grading scale used to assess AMI, although practical and commonly used, may not capture the full extent of motor inhibition or the subjective perception of neuromuscular control. In contrast, psychological constructs such as kinesiophobia are multifactorial, shaped not only by movement impairments but also by prior injury experience, personality traits, pain sensitivity, and external feedback from clinicians and peers [29,32]. Finally, it is possible that both AMI and kinesiophobia follow independent early trajectories after ACLR. AMI typically emerges immediately after surgery due to mechanical and inflammatory processes within the joint, whereas kinesiophobia may be more influenced by cognitive-emotional responses that evolve in response to persistent pain, frustration with rehabilitation, or perceived lack of progress. This temporal mismatch could explain the lack of statistical association in our cohort.

Together, these results suggest that although AMI and kinesiophobia are both important barriers to recovery, they may not be strongly interrelated during the initial postoperative weeks. Future research employing longitudinal designs and incorporating more granular neurophysiological and psychological assessments may help clarify whether these domains interact more meaningfully at later stages of rehabilitation.

The present study found no statistically significant differences in anterior tibial translation, as measured by the GNRB arthrometer, across varying grades of AMI. This result suggests that early postoperative mechanical knee laxity is not a determinant of clinically observable quadriceps inhibition within the first month after ACLR with hamstring autografts.

The rationale for exploring a potential relationship between anterior laxity and AMI lies in the hypothesis that increased joint instability may impair afferent sensory signaling, particularly from mechanoreceptors within the ligament and joint capsule [47,51,52]. This disruption in proprioceptive input has been proposed as a contributor to reflex inhibition of the quadriceps muscle [37]. However, our data does not support this hypothesis in the early postoperative context. The observed mean GNRB values across AMI categories showed minimal variability and no discernible pattern of association. These findings align with studies that have reported a lack of association between mild to moderate residual anterior laxity and neuromuscular outcomes. For example, Michel et al. found no significant link between graft laxity and quadriceps strength in the midterm follow-up after ACLR [42]. Similarly, Klouche et al. demonstrated that small differences in anterior translation, even when objectively measured using the GNRB device, did not correlate with clinical instability or functional deficits in low-demand activities [24]. One possible explanation for the absence of correlation is that the degree of anterior laxity observed in the early phase after surgery may not be sufficient to provoke the kind of proprioceptive disruption required to induce or exacerbate AMI. In our cohort, GNRB displacement differences were small and fell within clinically acceptable ranges for reconstructed knees. Furthermore, the intrinsic characteristics of the hamstring graft—such as initial tension, stiffness, and integration—may have mitigated any mechanical instability during this period.

It is also important to consider that AMI is strongly influenced by intra-articular inflammation and joint homeostasis, particularly within the first few weeks after surgery [17,19,40,50]. Factors such as synovial swelling, hemarthrosis, and nociceptive signaling may dominate the reflex inhibition pathways, overshadowing any subtle effects of anterior laxity. As noted by Sonnery-Cottet et al., early AMI is more consistently linked to these inflammatory and nociceptive triggers than to mechanical factors such as joint translation or rotational instability [17]. Taken together, these findings suggest that anterior knee laxity, at least within normative postoperative limits, is not a sufficient condition to influence the presence or severity of AMI in the early rehabilitation window. This further emphasizes the multifactorial nature of quadriceps inhibition, which likely arises from a complex interplay of biological, neurophysiological, and perhaps procedural variables rather than solely from biomechanical instability.

This study found no significant correlation between kinesiophobia, as measured by the TSK-11, and anterior tibial translation assessed by the GNRB arthrometer. These findings suggest that, during the early postoperative period (1 month after ACLR), perceived psychological fear of movement or reinjury does not appear to be influenced by the degree of anterior knee laxity as objectively measured. This result is consistent with several prior studies indicating that psychological outcomes in ACLR patients are not strongly associated with mechanical indicators of joint stability. Webster and Feller showed that athletes’ perceived readiness to return to sport is more strongly influenced by subjective factors—such as fear of reinjury, confidence, and personal expectations—than by objective knee laxity measured clinically or via arthrometry [30]. Similarly, Caumeil et al. demonstrated through cluster analysis that psychological distress and anxiety profiles in ACLR patients were not predicted by graft laxity or knee function scores [32]. The lack of correlation in our cohort may be partly explained by the multifactorial origins of kinesiophobia. While one might assume that patients with greater joint instability would develop more fear of movement, fear-avoidant behaviors are often shaped by broader psychosocial dynamics. Considering the injury history and other descriptive variables of the participants is essential to better understand the lack of significant associations between AMI, anterior knee laxity, and kinesiophobia observed in our study. Individual variability in factors such as previous injuries, chronicity, and rehabilitation adherence may modulate these relationships [29,31]. Future research should aim to incorporate comprehensive injury and clinical profiles to clarify these complex interactions. Moreover, patients in the early postoperative stage may not yet perceive or be aware of subtle differences in knee laxity, particularly in the absence of demanding functional challenges. At this point in recovery, most patients are still engaged in relatively low-intensity rehabilitation and are unlikely to test their knee in contexts where instability would be felt or interpreted as a threat. As such, objective anterior tibial translation—while critical for evaluating graft integrity—may not be relevant to patients’ psychological experience during this timeframe.

Finally, it is worth noting that our study cohort exhibited a relatively narrow range of anterior laxity values, consistent with normal early postoperative graft behavior. This limited variability may have further reduced the likelihood of detecting a meaningful association between mechanical laxity and kinesiophobia. In sum, our findings support the view that kinesiophobia in the early phases of ACLR rehabilitation is not mechanically driven and may require independent assessment and management, apart from biomechanical evaluations.

The timing of evaluation, limited to the 4–6 week postoperative window, represents both a strength and a limitation of the present study. This early phase is clinically relevant for initiating neuromuscular and psychological interventions, as it coincides with heightened neuroplasticity and motor relearning potential [26,27]. However, it may also be too premature to capture the full interplay between AMI, anterior knee laxity, and kinesiophobia. Psychological constructs such as fear of reinjury or avoidance behavior often become more pronounced later in the rehabilitation timeline, particularly during the return-to-sport phase [22,24,44]. Similarly, neuromuscular adaptations or compensatory strategies may evolve progressively, which could obscure early relationships between mechanical and psychological domains [41,42]. Moreover, although our analyses did not identify statistically significant associations, we observed subtle trends that may carry clinical relevance. Notably, patients with the most severe AMI grade (2b) demonstrated the highest TSK-11 scores, despite the small sample size. This may suggest that early neuromuscular inhibition could contribute to increased fear of movement in certain individuals. While this trend did not reach statistical significance, it highlights the potential utility of early screening for AMI severity as a predictor of psychological vulnerability. Future research should examine whether such early indicators can identify patients at risk for delayed recovery or suboptimal return-to-sport outcomes.

## 5. Limits

This study has several limitations that should be acknowledged when interpreting the findings.

First, the cross-sectional design precludes any conclusions about causality between AMI, anterior knee laxity, and kinesiophobia. The absence of temporal data limits our ability to determine whether these variables may influence one another at different stages of the postoperative recovery process.

Second, the sample size, while sufficient for detecting moderate correlations, was not large enough to robustly assess subgroup differences, particularly among the higher AMI grades (e.g., grades 2a and 2b). The small number of participants in these categories may have limited the statistical power to detect subtle effects. Potential influence of sex differences on neuromuscular and psychological responses following ACLR. Although men and women were included, the sample size did not allow for adequately powered sex-specific analyses. Given known sex-related differences in ligamentous laxity, pain perception, and psychological factors, future studies should aim to explore these variables more explicitly.

Third, the clinical grading scale used to assess AMI, although grounded in previous literature [8,9,10,33], has not been formally validated. Its semi-objective nature and potential inter-rater variability represent limitations. Future studies should consider the use of more objective neuromuscular evaluation methods, such as electromyography or central activation ratio, to better quantify quadriceps inhibition.

Fourth, although our total sample size met the requirements from a priori power calculations, the uneven distribution across AMI grades, particularly the small number of patients in grades 2a (*n* = 9) and 2b (*n* = 2), may have limited the statistical power for detecting intergroup differences. This limitation should be considered when interpreting the subgroup analyses. Besides mechanisms of injury, such as contact versus non-contact or training versus competition context, which were not systematically recorded in this study, the literature consistently shows that the majority of ACL injuries in active populations occur through non-contact mechanisms involving sudden deceleration, cutting, or landing tasks. This context supports the clinical relevance of investigating neuromuscular and psychological factors in this population.

Fifth, the study sample was composed exclusively of young and physically active individuals with high pre-injury activity levels, which limits the generalizability of the findings. The observed relationships—or lack thereof—between AMI, anterior knee laxity, and kinesiophobia may differ in older patients, those with lower baseline fitness, or those with less demanding functional goals. Future research should include more diverse populations to assess whether these variables interact differently across age groups, activity levels, and rehabilitation contexts.

Finally, although we included a validated measure for kinesiophobia (TSK-11), other psychological constructs such as pain catastrophizing, self-efficacy, or perceived function were not assessed and may play an important role in early postoperative recovery [53,54,55].

Future studies should consider longitudinal approaches to track the evolution of AMI, laxity, and psychological responses over time. Incorporating objective neuromuscular assessments and a broader range of psychological outcomes would enhance our understanding of the multifactorial barriers to recovery. Identifying patient subgroups at higher risk of delayed progression may also support the development of individualized rehabilitation strategies that target mechanical, neuromuscular, and psychological domains in parallel.

## 6. Futures Perspectives

Building on our findings, future research should prioritize larger, sex-balanced cohorts to elucidate potential sex-specific recovery trajectories. Longitudinal designs are necessary to capture temporal changes in AMI, knee laxity, and kinesiophobia throughout rehabilitation. Moreover, integrating objective neurocognitive and neuromuscular assessments, such as electromyography and reaction time measures, may enhance understanding of underlying mechanisms and inform tailored intervention strategies. 

## 7. Conclusions

This study highlights the complex and multifactorial nature of early postoperative recovery following ACL reconstruction. The heterogeneous clinical profile of patients may limit the generalizability of the findings, underscoring the need for personalized assessment and intervention. Practically, early identification and targeted management of arthrogenic muscle inhibition may optimize quadriceps function; monitoring anterior knee laxity remains crucial for joint stability assessment; and addressing kinesiophobia through psychological support could facilitate return to activity. Future rehabilitation programs should integrate these dimensions to improve patient outcomes.

## Figures and Tables

**Table 1:** Participant’s characteristics.

Variable	Mean	Standard Deviation
Women	15	
Men	41	
Age (years)	26.48	5.71
Height (cm)	175.88	9.79
Weight (kg)	81.16	19.66
BMI	26.08	5.00

Note: BMI; Body Mass Index.

**Table 2:** TSK-11 and GNRB scores according to the AMI grade.

		AMI GRADE					One-Way Anova Test/Kruskal-Wallis H Test *p* Value
		0 (*n* = 27)	1a (*n* = 11)	1b (*n* = 7)	2a (*n* = 9)	2b (*n* = 2)	
TSK-11 (score)	Mean	39.15	41.09	39.00	36.78	45.50	0.327
	SD	6.09	6.01	6.14	5.65	4.95	
GNRB (mm)	Mean	0.96	0.97	0.79	0.67	− 0.45	0.182
	SD	0.79	0.56	0.77	0.53	0.78	

Note: SD; standard deviation, AMI; Arthrogenic Muscle Inhibition, TSK; Tempa Scale Kinesiophobia.

## Data Availability

The data supporting the findings of this study are available from the corresponding authors upon reasonable request.
